# An Event Driven Hybrid Identity Management Approach to Privacy Enhanced e-Health

**DOI:** 10.3390/s120506129

**Published:** 2012-05-10

**Authors:** Rosa Sánchez-Guerrero, Florina Almenárez, Daniel Díaz-Sánchez, Andrés Marín, Patricia Arias, Fabio Sanvido

**Keywords:** identity management, privacy, user-centric, federation, revocation consent, delegation, health care, event, theory queue

## Abstract

Credential-based authorization offers interesting advantages for ubiquitous scenarios involving limited devices such as sensors and personal mobile equipment: the verification can be done locally; it offers a more reduced computational cost than its competitors for issuing, storing, and verification; and it naturally supports rights delegation. The main drawback is the revocation of rights. Revocation requires handling potentially large revocation lists, or using protocols to check the revocation status, bringing extra communication costs not acceptable for sensors and other limited devices. Moreover, the effective revocation consent—considered as a privacy rule in sensitive scenarios—has not been fully addressed. This paper proposes an event-based mechanism empowering a new concept, the *sleepyhead credentials*, which allows to substitute time constraints and explicit revocation by activating and deactivating authorization rights according to events. Our approach is to integrate this concept in IdM systems in a hybrid model supporting delegation, which can be an interesting alternative for scenarios where revocation of consent and user privacy are critical. The delegation includes a SAML compliant protocol, which we have validated through a proof-of-concept implementation. This article also explains the mathematical model describing the event-based model and offers estimations of the overhead introduced by the system. The paper focus on health care scenarios, where we show the flexibility of the proposed event-based user consent revocation mechanism.

## 1. Introduction

Sensors are the primary source for data collection. Often the collection process and the collected data have to be taken into account when defining the privacy policies of systems. Privacy has a different meaning for every individual: it is common to find differences among users when it comes to what should be protected and how. Thus this is a very complex and subjective concept that relates the sensitiveness of the information owner to the context in which the information is used. In a digital context, users' information is extensively collected and distributed to provide new added value services and to improve availability. Whereas these new services have a positive impact on users' life, they also bring privacy problems. A good example are social networks. Pervasive computing and social immersion have made users active broadcasters of their own life producing information streams and multimedia that will be eventually published in a social network. Users are expected to deal responsibly with the privacy agreements exposed by the participants in a digital transaction, but the complexity of some agreements and the increasing number of participants overwhelm users. In the end, the privacy issues that rise when distributing user information are considered by the users themselves as minimal tradeoffs in comparison to the benefits of accessing services. This is extremely worrying, specially for young people, since despite they have born into technology (*i.e.*, “digital natives”), they are not conscious about the consequences when it comes to spread their personal information all over the Internet.

The motivation behind this problem is the lack of comprehensive privacy frameworks. Individuals want to have control of their information since the improper and unsecured management of their information may lead to attacks, frauds, and identity misuse, as identity information can be exploited whenever authentication and authorization based on those identity attributes are required. In [1], an architecture that allows to identify different levels of Quality of Privacy based on user context is proposed.

Often sensor networks, and specially wireless sensor networks, use encryption to provide data protection and integrity, and key distribution has deserved important efforts, both for asymmetric and symmetric keys [2,3]. There are not so many works in authorization, and the application of flexible role-based or attribute-based authorization schemes has not been sufficiently addressed. One of the main problems is that rights revocation requires the execution of complex processes, which are simply not acceptable for limited sensor networks. Today identity is approached in a different way. Modern digital identity is not just authentication or authorization data but a collection of attributes that embraces different kind of information related to the user: attributes subject to verification (as age or gender), history of previous interactions, preferences, reputation information… Service providers employ Identity Management (IdM) Systems for managing the increasing volume of information. IdM frameworks provide technical means for sharing users attributes among different participants typically between an Identity Provider (IdP-the entity retaining user's data) and Service Providers (SP-information consumers). IdM technology has developed quickly in the last decade and it has been widely adopted by popular service providers since it has shown itself to be a reliable and efficient way to handle users information. IdM systems mediate in every users' information exchange, so they are the preferred target where to deploy privacy solutions. For instance, in emerging mobile health applications based on Internet of things, IdM enables to preserve privacy by ensuring anonymous consultation from external doctors [4]. As it will be discussed in this article, there are several approaches to identity management being the most popular the federated and user-centric approaches. These approaches provide many services as the popular Single Sign On (SSO) across multiple trusted domains. SSO allows users of one domain to securely access resources of another domain seamlessly, requiring no redundant login processes. Both approaches have benefits and shortcomings, for instance, the federated model has scalability issues which the user-centric model solves, but both of them can be used for a better privacy management.

Current IdM systems are not ready to cope with some aspects of privacy. Specifically, the user consent revocation is not covered by any of the aforementioned identity management approaches. This fits with the privacy view as control over the use and flow of one's personal information [5]. Revoking consent allows users grant or withdraw consent of specific actions over data to certain individuals. So this mechanism is useful to enforce user's role in the task of preserving her privacy. This property is part of the privacy rules described by the HIPAA (*Health Insurance Portability and Accountability Act*) [6] for health, OECD (*Organization for Economic Cooperation and Developments*) principles and GLBA (*Gramm*–*Leach*–*Bliley Act*) for financial institutions, and the COPPA (*Childrens Online Privacy Protection Act*) for parental control. E.U. citizens also have the right to revoke consent. This is not in the European Union, where a person's consent cannot be given prospectively and where consent must be fully informed [7]. The E-Privacy Directive [8] addresses particular concerns in the use of electronic communications to deal with personal data; however it alone does not provide an adequate revocation solution, conferring as it does only limited rights on individuals to prevent types of processing by withdrawing consent to such processing [9]. For instance, a parent may revoke his consent to allow a social network to use his child's personal information. Nevertheless, the research in this field is still in its early stage.

In this article, we focus on the privacy concept within identity management systems (IdM) in ubiquitous environments. Particularly, we have selected health care scenarios since they are among the most sensitive scenarios. Our privacy-aware identity system implements an effective consent revocation with an innovative event management that can be used in other scenarios. For this purpose, we assume that the development of patients care can be broken down into events. These events describe a specific situation and can be related to some participant entities. We propose a delegation protocol, which issues a *sleepyhead credential* containing user's attributes and access privileges that have been granted beforehand but are kept latent. To use these attributes, an activation process is necessary. Our solution proposes events to awake dormant privileges or part of them and incorporates several new features that allow better scalability. For instance, emergency services are the entities which manage trust indirectly by triggering events that will be used to determine which participants can access to user's medical information. Moreover, we provide a mathematical model that describes and evaluates the processes of event arrivals and notification message management.

The rest of this paper is structured as follows: Section 2 presents background regarding the main privacy principles and rules and current identity management approaches, identifying the advantages and drawbacks of each one in terms of privacy. Section 3 highlights the importance of revoking consent and provides a comparative analysis of the privacy support in identity management systems. Section 4 explains our proposal to enhance privacy in health care scenarios. A mathematical model, which describes the event-driven system behavior, is also illustrated. Then, Section 5 introduces implementation issues and shows simulation results concerning the event engine. In Section 6, we give an overview about related work. Finally, Section 7 summarizes the presented work and presents the main conclusions and future research lines.

## 2. Background

As mentioned before, privacy is complex to handle and needs to cope with different sensitivities that depend, among others, on the context in which the information is used. Privacy comprises several principles and rules such as anonymity, pseudonymity, unobservability, unlinkability, and revocation consent. These concepts might have different definitions in the literature [10]. This section firstly defines such concepts. Afterwards, we discuss how these concepts are addressed in identity management systems, as well as privacy policies languages.

### 2.1. Principles and Rules of Privacy

- ***Anonymity*** can be defined as the state of being not identifiable within a set of subjects or entities, also called the *anonymity set*. Another definition provided by the Common Criteria [11], asserts that this property ensures that a user may use a resource or service without disclosing the user identity. Cryptographic techniques, such as encryption, do not guarantee anonymity since an observer could analyze traffic, eavesdrop the sender of the message and follow the message up to the receiver, establishing certain relationships without having access to the unencrypted message. Therefore, IdM systems must provide additional mechanisms, such as opaque identifiers to prevent such inference.
- ***Pseudonymity*** is the use of pseudonyms as identifiers. An advantage of pseudonymity technologies is that accountability for misbehavior can be enforced. Thus, this enables IdPs that can link identifiers to real identities to make appropriate decisions when a user commits a crime or offense in an IdM scenario.
- ***Unlinkability*** ensures that a user may consume multiple resources or services without letting other entities to link these multiple resource or service accesses together. In particular, this property allows users to interact with multiple organizations (SPs or IdPs), each of them able to map a user to a given identity, using different identities. Moreover, IdM systems should provide mechanisms to prevent collaborating organizations from linking a given user profile at one organization with the same user profile at another. While it is relatively easy to let users create and maintain multiple identities for themselves, ensuring that these identities remain unlinkable is not straightforward. In particular, there is always a risk since patterns of usage and attribute values might leak enough information to link the identities of a given user.
- ***Unobservability*** permits a user to access resources or services avoiding other entities, especially third parties, to observe that the resource or service is being used. Regarding identity management, traffic analysis is a well-known example, which tries to violate this principle.
- ***Revocation consent*** allows users to withdraw consent of specific actions over data to certain individuals. So this rule is useful to enforce user's role in the task of preserving her privacy.

### 2.2. Current Identity Models

According to Josang *et al.* [12], the fundamental privacy protection principle is that exposure of personal information should be minimized. If we transfer this concept to identity management approaches, this means that the fewer parties involved in the management of the identity information the better. Nevertheless, achieving a good degree of privacy implies observing every aforementioned privacy principle. Furthermore, although the property of anonymity is one of the four principles of privacy, it would be desirable that IdM systems support mechanisms to break the anonymity of a user for the purpose of analysis or evidence under certain circumstances (e.g., a malicious user, lawful interception).

For clarity, we introduce here the main actors in an identity management scenario. (i) The Principal, or the End User, who has a particular digital identity and interacts (usually via an user agent) with SPs. (ii) the Service Provider (SP), which provides services and takes decisions about a particular subject based on the identity information provided by (iii) the Identity Provider, that authenticates users, manages identity information and shares identity information with various SPs upon user request.

#### 2.2.1. Federated Identity Model

**Figure 1.**
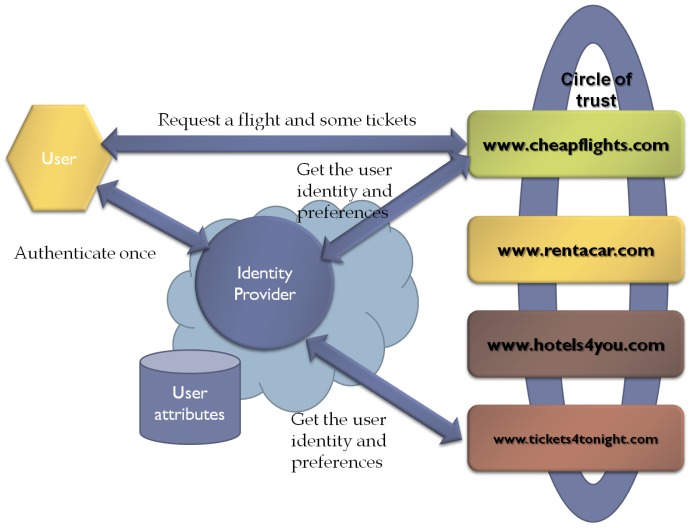
Federated model scenario. A user, after a successful authentication, can access services from any service provider within the circle of trust. For instance, booking a flight, then renting a car, and finally buying tickets for a show. Note that the IdP stores identity information on behalf of the user.

The identity federation model can be defined as a set of standards, technologies and agreements that enable SPs to recognize user identities and entitlements from other SPs or IdPs. Thus, this approach is based on groups of SPs and IdPs that have a pre-existing mutual trust relationship. Consequently, specifications, such as Security Assertion Markup Language (SAML) [13], recommend using Public Key Infrastructure (PKI) [14] for establishing trust relationships. Regarding the terminology of Liberty Alliance (ID-FF) [15], the above groups are called members of the *circle of trust* (see Figure 1).

Federated models bring user attribute exchange, user account provisioning, entitlement management and personalized service provisioning. Nevertheless, as far as usability and scalability are concerned, this model has several drawbacks. For instance, it adds further legal and technical complexity, since to be part of the circle of trust, an entity would need to sign a legal agreement. In addition, federated model presents scalability issues when deployed in dynamic open environments due to rigidity and staticity of the agreements between federated organizations. A comparative analysis of the underlying trust mechanisms of the current frameworks for federated identity management can be found in [16].

From a privacy perspective, the federated identity approach has both advantages and disadvantages. Regarding its advantages, it allows users to have multiple identities within a given domain. Similarly, the federated model enables an entity to have different identities or identifiers in different domains. These features make it possible for a single identity to have different identifiers in different domains, e.g., as patient in a health care domain, as employee or student in another domain, *etc.* Moreover, from the SP perspective, the identifier mapping permits different SPs to refer to the same user through different identifiers. Whereas the IdP needs to know the “real world” identity of a user, this user identity can be anonymous for a specific SP, which provides additional privacy protection. However, it must be noted that users never participate in the trust establishment process so they need to believe that the IdP will behave honestly.

The main drawback of this kind of federated identity models is that the privacy protection depends on the privacy policy and the adherence of the IdP or SP to the policy, which can be a threat. For instance, different SPs could be able to match personal information of the same user because of the mapping between identifiers. In order to prevent this problem, identity frameworks such as SAML and Liberty, advise the use of pairwise, directional opaque identifiers.

#### 2.2.2. User-Centric Identity Model

The user-centric model places the user in the middle of a transaction. Thereby, this approach gives users total control over their identities, as well as control over authentication and attribute exchange processes. In this way, the user is no longer aside of the trust establishment process. However, this does not mean that users should approve every transaction, but that data always flow through the user's identity agent. This approach indeed empowers users and follows better than the federated model the philosophy of *minimal disclosure* defined by Josang. Moreover, from the usability perspective, the user-centric identity model solves scalability problems and provides similar services as SSO, whereas is compatible with the federated model.

**Figure 2.**
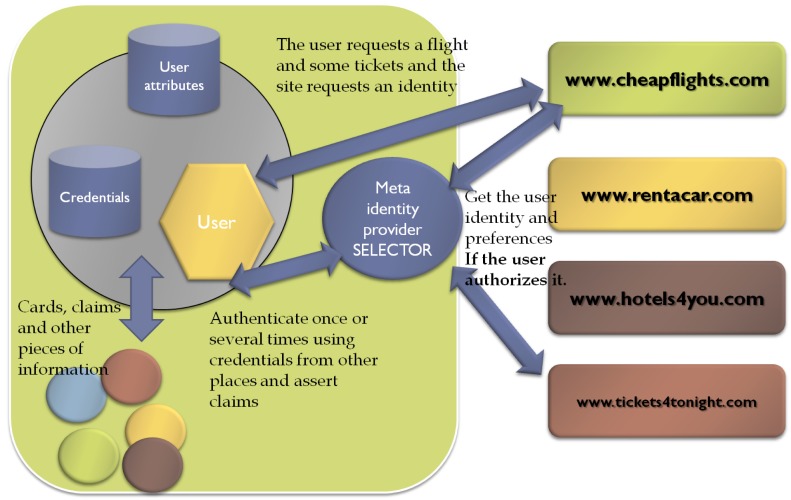
User centric model. A user can access services from any service provider accepting his/her credentials. For instance, booking a flight, then renting a car and finally buying tickets for a show. Note that the information is provided always by the user.

With regard to privacy, this model has both advantages and drawbacks. It introduces the concept of *meta-idp*, which allows users to assert several kind of claims: *user-generated* and *provider-generated* claims. These user electronic identities are typically stored in user's equipment, such as his mobile phone. User-centric identity technologies, such as InfoCards [17], allow users to select among their multiple identities through identity selectors to identify herself to a service. Regarding identity selectors, in [18] two types of information cards are specified: *Personal* or *Self-Issued* (claims about the user itself, e.g., phone number, e-mail address, web address); and a *Managed Information Cards*, issued by IdPs. The latter can be auditing, non-auditing, or auditing-optional to accommodate the needs of different business models. The identity cards are metaphors of real id cards whereas the identity selector mimics a wallet. However, it is worth mentioning here that, in the case of *provider-generated* claims, the user must rely on the IdP honesty, as occurred in the federated model (see Figure 2).

The main disadvantage of user-centric approach is that it requires a complex design in order to avoid privacy and trust issues with authentication and attribute verification. In order to assist the reader in understanding this aspect, we provide the following example. If we consider a real world example in which Bob may show his driver licence to a bartender to prove he is above the legal drinking age, we can see that Bob is able to use his Id card without the Id card issuer's knowledge. However, if we transfer this example to a user-centric scenario, trust and privacy problems emerge, because no SP is obliged to believe Bob when he asserts that he is old enough to legal buy alcoholic beverages. In this sense, it is necessary that a trusted third party corroborates the above statement by using a provider-generated card.

## 3. Motivation

Current identity management technologies are not ready to cope with user consent revocation in an appropriate way. This is a relevant issue with regard to privacy-enhancing mechanisms, especially in sensitive scenarios when sensitive data and profiles are shared. For instance, in health, economy and/or parental control scenarios. In a health care scenario, the system must protect user's privacy and allow authorized entities (including humans) to access medical records conveniently. Moreover, privileges permitting access to user attributes should be revoked in an effective way.

### 3.1. Lack of Appropriate Revocation in Current Identity Management Systems

Nowadays, the several federated and user-centric identity management frameworks have not addressed this privacy rule. The privacy support of the aforementioned technologies is analyzed below. SAML is a federated specification, which supports two types of identifiers to refer to users: *transient* or *one-time identifiers* and *persistent identifiers*. On the one hand, transient identifiers ensure that a user anonymously accesses a service during SSO process, since these identifiers are created for use during a session and they are destroyed at the end. Thus, correlation between identifiers is avoided. On the other hand, the persistent identifiers provide a persistent federation and remain active until they are explicitly deleted. The permanent federation implies an account linkage process, which relates two accounts associated to a user in different SPs. Note that it is recommended to use different pseudonyms for each SP, in order to avoid different SPs belonging to the same federation to infer user behavior.

SAML supports partial anonymity in the sense that the IdP itself is able to know which user corresponds to each identity. Indeed, SAML does not provide a solution from preventing IdPs from tracking user's visits to SPs. Regarding privacy policies, this technology allows to obtain a principal's consent or describe specific attributes to satisfy requirements to preserve privacy within a health care community, through the XSPA-SAML profile [19]. Nevertheless, SAML standard states that privacy must be considered, but concrete decisions are left to the implementors.

With regard to the Liberty Alliance federated model, it defines an Identity Governance Framework (IGF) [20], which enables the creation of policies or contracts between an Attribute Provider (AP) and a SP. Therefore, IGF includes two XML syntaxes: Attribute Requirement Markup Language (ARML) and Attribute Authority Policy Markup Language (AAPML). Moreover, IGF defines basic privacy constraints such as usage, storage, propagation and display of identity data. Thus, an attribute provider creates statements to access and use protected attributes. At the same time, a SP may specify whether the requested attributes will be discarded after usage. Furthermore, the SP could request to modify the data or forward it to another SP. However, in [21], Liberty proposes a multi-level policy approach, which does not consider any specification or rules for storing user preferences in a manner that would facilitate the SPs to match the privacy policy levels in the attribute request with the levels in user's preferences. As SAML, Liberty offers *long-term* and *one-time* pseudonyms. Correspondingly, it must be noted that this specification only allows a user to have one long-term pseudonym per SP to prevent user tracking across different transactions. This is a big limitation. In addition, it does not protect against SPs cooperating to share user pseudonyms in order to track users behavior. In order to overcome these problems, a set of rules and recommendations are proposed in [22].

In the case of InfoCards, this technology includes authenticated anonymity and pseudonymity, as well as the ability to express privacy policies of SPs or Relying Parties (RPs). This user-centric framework is characterized by defining a message flow that eliminates direct communication between the IdP and the SP. Moreover, InfoCards allow the identity selector to encrypt the SP identity to prevent the IdP from learning the SP identity when it receives a request for a token. Note that, this identity selector applies user-centric principles in collecting user consent. Both features together are necessary to ensure that an IdP cannot learn which SPs visit a given principal. The SAML Enhanced Client Proxy profile (ECP) is similar, but currently it only has the first characteristic. However, some IdPs may require knowledge of the RPs identity before issuing a requested token, or even if the IdP cannot learn the visited SPs, user profiling is possible by colluding parties. Regarding OpenID, privacy considerations are not addressed in the main specification and SSO can be performed between previously unknown parties without any configuration. Thus, there is no trust model, the protocol operates in accordance with the *trust-all-comers* philosophy. Although for some services requiring no verification this model may be sufficient, this mechanism is too simple and unsafe for many other applications, leading to privacy breaches. Nevertheless, an OpenID extension called PAPE (Provider Authentication Policy Extension) [23], provides the means for a RP to request previously agreed upon authentication policies being applied by the OpenID Provider and for an OpenID Provider to inform an RP what policies will be used. Therefore, the decision to trust can be based on the knowledge of the authentication mechanism employed. Hence, with this user-centric framework, RPs must decide for themselves which providers are trustworthy, being able to enforce policies to the OpenID Provider's response.

Table 1 summarizes the main privacy features grouped by IdM models. The technologies that have been analyzed handle privacy by means of pseudonyms which can be transient or permanent. The only exception is OpenID, which follows the *trust-and-accept-all-comers* principle and privacy is not addressed. Moreover, it must be noted that InfoCards and SAML ECP profile address better the principle of minimal disclosure. Current identity frameworks support partial anonymity, since authorities, as the IdP, provides obfuscated identifiers. We want to stress the importance of privacy policies, since they are the basic means that allow users to understand privacy implications in terms of attribute exchange or delegation between different security domains. Though privacy policies are critical for users to give their consent, they are often poorly defined, complex to implement, or simply out of scope of the specifications.

### 3.2. The Need for a Time Independent Revocation System

The problem of revoking consent is covered by none of the aforementioned IdM technologies. Thus, if personal data have been already shared, the effective revocation of consent implies an important challenge to address. For instance, it requires dynamic updates to sticky policies.

Consider that Bob, a doctor, has been assigned to Alice for pre-diagnose. He should be authorized to access Alice's medical records (*i.e.*, blood test) but after that evaluation, Bob privileges should be revoked. If the revocation is based on time, as PKI-based solutions, Alice should wait some time until Bob privileges expire to be sure he is no longer able to access her records. In this case, the time window after Bob finishes the pre-diagnosis until the privileges are revoked is a window of opportunity Bob has, to access Alice records without explicit permission. This fact complicates the accounting, since after the first legal access, further (illegal) accesses to records will be related to the initial authorization.

To overcome this problem in time based revocation, the validity time of a given privilege set can be set to a very short period of time, so the opportunity window is reduced. On the contrary, if the token duration is longer than necessary, user's sensitive information may be exposed to entities which should not have access to that information during that time.

As a result of this previous discussion, our motivation is to provide a flexible event-based user consent-revocation mechanism. So, in the previous scenario, after Bob finishes the pre-diagnosis, a new event is issued (*i.e.*, needs surgery) and Bob privileges are automatically revoked.

## 4. Enhancing Privacy: A Hybrid IdM Event-Driven Approach

In this section we present a hybrid identity model, because in health care scenarios attribute exchange and delegation process cannot be completely user-centric, since in cases of critical accidents the user cannot be able to give her consent. On the other hand, federated models raise privacy concerns since medical records may be available to every entity within the *circle of trust*, even if there is no emergency. Hence, we propose a hybrid model, which allows users to configure and track access to their medical records while the identity providers are the entities in charge of storing and managing users' *sleepyhead credential*. Such *sleepyhead credential* is distributed through a delegation protocol for time-independent revocation. The *sleepyhead credential* contains user's attribute identifiers (*i.e.*, her medical history), as well as access privileges, which have been granted to beforehand but are latent. Thus, to use the aforementioned attributes or privileges, an activation process is necessary. Particularly, within health care scenarios, we can model patients life cycle as event-driven [24,25]. Events are fired by trusted entities when specific circumstances are met, and routed to required entities. We propose using these events to awake the dormant privileges or part of them. Moreover, in order to prevent unauthorized access, we require some entities to use a **Privacy Engine**, responsible for analyzing events and activating the strictly needed attributes and privileges for each event. The following sections explain the assumptions made during the design of our hybrid IdM event-aware as well as the proposed system and the way in which the delegation protocol, along with the event engine, allows to revoke user consent. Finally, we introduce a mathematical formulation of the event model.

### 4.1. Hypotheses

This section describes the assumptions on which our *Sleepyhead Credential*-based delegation protocol has been built. We assume the existence of an event engine, which follows a notification model based on the SIP-Specific Event Notify [26] specification to send events to entities (by means of broadcast or unicast to registered entities). We assume that the entities persisting the medical records act as IdPs and those requesting access to medical records acts as SPs. SPs, as hospitals, emergency services and even individuals, as doctors, can issue events that will be routed to appropriate medical record holders (IdPs) in order to unblock medical records. We assume that events follow well known workflows usually triggered by well known trusted entities as emergency services. Moreover, events are achieved allowing rogue entities to be traced if they interfere in the process. It should be noted that SPs and IdPs can take the role of subscribers and notifiers, in some circumstances, either subscribing to different events or notifying them. In order to clarify this last aspect, consider that some parts of the patient's medical history reside in different IdPs and depending on the required treatment, it is necessary to consult several parts of the medical record, thereby an IdP can act as both client (subscriber) and server (notifier). Besides, each entity can be subscribed to multiple types of events, as well as each event type can be attended by several notifiers.

**Figure 3.**
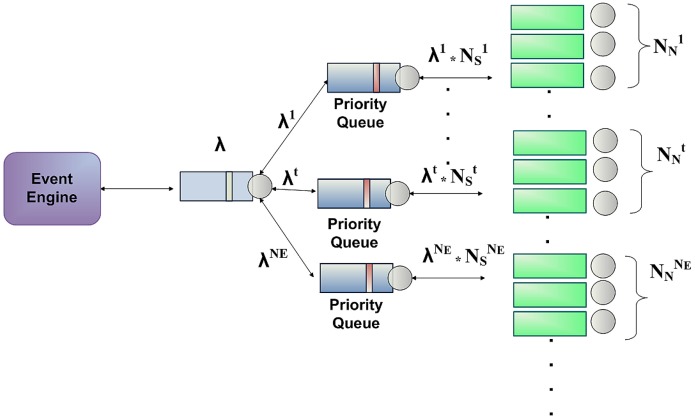
Event queueing system.

Patients life cycle modeled as event-driven, as well as Poisson distributed arrival rate of health care events and service rate (*i.e.*, patient arrivals at an emergency service) are widely adopted and well-known by existing research work in the literature, such as [27–29]. Therefore, we suppose that the arrival process of the events to our system conforms to Poisson distribution with parameter λ and the processing time of these events conforms to exponential distribution, then from the queueing networks result [30], the outgoing process of NOTIFY messages is also Poisson distribution (see Figure 3).

Concerning security, communication confidentiality should be granted specially for sensitive environments like health care. For that reason we assume the use of HTTPS with mutual authentication to handle message exchange. We assume as well that HTTPS certificates have been correctly issued and distributed. Furthermore, note that it is necessary to take into account security considerations regarding SIP SUBSCRIBE and NOTIFY messages, given the high sensitivity of health care data considered in the proposal. Therefore, both subscription and notification messages must be authenticated and authorized, for instance to prevent the participating entities from subscribing multiple times or redirecting the subscription of their neighbor either intentionally or accidentally. In this sense, SIP can use different security mechanisms such as HTTP Digest or TLS. We recommend TLS for secure and encrypted SIP communications. Besides, all users utilize transient identifiers in order to preserve their anonymity while enabling IdPs accountability enforcement in case of user's misbehavior, according to the main principles of privacy specified in Section 2. A Public Key Infrastructure (PKI) can be easily used to support secure communication channels. Finally, we assume an underlying trust relationship based on PKI for entities belonging to different domains.

### 4.2. SAML-Compliant Sleepyhead Credential

The *Sleepyhead Credential* (SC) has been defined as a new SAML assertion according to the SAML proposal for delegation information defined in [31]. Thus, the *Sleepyhead Credential* is created with the following tuple:

(1) $$\text{SC}=\{\mathrm{Att}P_{1},\mathrm{Att}P_{2},\ldots ,\mathrm{Att}P_{n}\}$$

where each component *AttP_i_* represents attributes and access privileges, which have been granted to any entity beforehand but remains latent until activated. A *Sleepyhead Credential* is composed of fields, including the following elements for delegation restriction: EventFilter, defines filters that will be used by the *Privacy Engine* to analyze the received events and decide whether any attribute(s) may be activated; TrustedEventSources, contains entity names whose events activate the credential; EntityMedicalRepository, specifies the location and distribution of attributes and medical records. The SAML assertion is defined as follows:

<complexType name=“DelegationRestrictionType”>
<complexContent>
<extension base=“saml:ConditionAbstractType”>
<sequence>
<element ref=“del:EventFilter” maxOccurs=“unbounded”>
<element ref=“del:TrustedEventSource” maxOccurs=“unbounded”>
<element ref=“del:EntityMedicalRepository” maxOccurs=“unbounded”>
</sequence>
</extension>
</complexContent>
</complexType>

This Sleepyhead Credential Assertion is exchanged using the SAML “Authentication Request Protocol”. Therefore, the SAML requests and responses are exchanged using the bindings defined in the specification and they are compliant with the rules defined for extending the schema.

Eventually, the IdPs will be the entities responsible for storing and managing the *sleepyhead* credentials, since as we have mentioned before, in cases of serious accident, the user may not be able to provide his credentials.

### 4.3. Privacy Engine

It is responsible for activating the latent attributes and privileges (following the principle of *minimal disclosure*) depending on the different event filters and the defined privacy policies. To this end, it analyzes the different elements which compose each event (*i.e.*, issuer, situation, degree of severity), as well as their purposes (*i.e.*, health care treatment, operation, emergency treatment) and applies the corresponding privacy policy. This policy includes the set of consent directives and other privacy conditions (*i.e.*, object filtering, user, role, purpose) that constrain enforcement.

On the other hand, the *Privacy Engine* includes an audit service for events, attribute activation, and access control decisions. It monitors how user data is being used without compromising user's identity. To accomplish this, the fields that are logged must be able to show the auditor what information about the user is being accessed without divulging the actual information. Note that this audit service itself will not physically prevent privacy breaches from occurring, but it can act as a deterrent and allow individuals and regulatory bodies to monitor how data is being shared, in order to prevent from *linking* and *traffic analysis attacks*.

### 4.4. Mathematical Formalization of the Event-Based Model

The purpose of this section is to mathematically describe how our event-driven system operates. In this sense, Markov's chains provide support for problems involving decision on uncertainties through a continuous period of time. Specifically, Markov models consider the patients in a discrete state of health, and the events may represent the transition from one state to another. Moreover, these approaches enable to model repetitive events and time dependence of probabilities [32]. So, we assume that events arrive to the system according to a homogeneous Poisson process with rate λ and to be consistent with an exponential distribution. A summary of the definitions and parameters that are used in this section is shown in Table 2. Equation (2) defines the set of entities of the system (SPs or IdPs), Equations (3) and (4) denote the notifiers and subscribers of the system, respectively. Finally, Equation (5) describes the set of events that can be triggered by the system:

(2) $$ES=\{es_{1},es_{2},\ldots ,es_{N_{ES}}\left|ES\right|\}$$

(3) $$N=\{n_{1},n_{2},\ldots n_{N_{N}}\left|N\right|\}$$

(4) $$S=\{s_{1},s_{2},\ldots s_{N_{S}}\left|S\right|\}$$

(5) $$E=\{e_{1},e_{2},\ldots e_{N_{E}}\left|E\right|\}$$

where

(6) $$\begin{array}{l} N_{N},N_{S}\leq N_{ES}\\ N_{N}+N_{S}\leq 2N_{ES}\\ N_{N}^{t}=p_{N}^{t}N_{N}\leq N_{N}\\ N_{S}^{t}=p_{S}^{t}N_{S}\leq N_{S} \end{array}$$

Furthermore, being {*N*^1^,…, *N^t^, N^u^, N^v^*,…, *N^NE^*}a subset of *N*, i.e, notifier subsets of events *e*_1_, *e*_t_,*e*_u_,*e*_v_, *etc*., then

(7) $$\left|\overset{N_{E}}{\underset{t=1}{\cup }}N^{t}\right|=\sum_{t=1}^{N_{E}}\left|N^{t}\right|-\sum_{t,u}\left|N^{t}\cap N^{u}\right|+\sum_{t,u,v}\left|N^{t}\cap N^{u}\cap N^{v}\right|+\ldots +{\left(-1\right)}^{N_{E}-1}\left|\overset{N_{E}}{\underset{t=1}{\cup }}N^{t}\right|$$

(8) $$N_{N}=\sum_{t=1}^{N_{E}}\left|N^{t}\right|-\sum_{t=1}^{N_{E}}\left|\cup N^{t}\right|$$

Similarly with subscribers, it can be established that, if {*S*^1^,…,*S^N_E_^*} are subsets of *S*, then

(9) $$N_{S}=\sum_{t=1}^{N_{E}}\left|S^{t}\right|-\sum_{t=1}^{N_{E}}\left|\cup S^{t}\right|$$

Also, we define *M^t^* as the message size to be transferred (considering the overhead introduced by the protocol) when a event of type *e_t_* is delivered. Moreover, λ*^t^* and λ*^t,k^* are the *e_t_* event arrival rate and *e_t_* event notification rate for notifier *n_k_*, respectively. $\lambda_{j}^{t}$, $\lambda_{j}^{t,k}$and $P_{en,j}^{t}$ are the rate of events of type *e_t_* that arrives to entity *e_j_*; and the rate and percentage of events of type *e_t_* notified by the *n_k_* notifier that arrive to the entity *e_j_*, respectively Thus, we define the rates as:

(10) $$\lambda_{j}^{t,k}=P_{en,j}^{t}\lambda^{t,k}$$

(11) $$\lambda_{j}^{t}=\sum_{k=1}^{N_{N^{t}}}\lambda_{j}^{t,k}$$

(12) $$\lambda^{t}=\sum_{k=1}^{N_{N^{t}}}\lambda^{t,k}$$

(13) $$\lambda =\sum_{t=1}^{N_{N_{E}}}\lambda^{t}$$

Hence, an entity can be subscribed to several notifiers. Once an *e_t_* event happens, the corresponding NOTIFY messages are scheduled to be sent to all the entities which are subscribed to this type of event. Figure 3 illustrates the process when an *e_t_* event is received, $N_{S}^{t}$ NOTIFY messages are generated. Thus, $\lambda^{t}N_{S}^{t}$ is the arrival rate for messages notifying *e_t_* events and there are $N_{N}^{t}$ notifiers or servers. In addition, as explained before, the service time for an *e_t_* event NOTIFY message is assumed to be exponentially distributed with mean 1/*μ*. Therefore, if we consider a queueing system that has *c* servers (being $c=N_{N}^{t}$ with *K* finite capacity, Poisson distributed incoming rates and exponential distributed service rates, this queueing system can be denoted by *M/M/c/K* [33]. It is worth noting that, when arriving health care events are placed in different queues, each will have a different service priority. We propose a priority discipline for different categories of events and then a first-in-first-out discipline for each category. For instance, urgent events will have a higher priority than non-urgent events, since an emergency department should treat patients with life-threatening injuries before others. Moreover, knowing the average frequency of events per period, it is possible to derive the probability of a certain number of events to arrive to the system for a given period. This is derived using Poisson's probability distribution:

(14) $$P_{n}=\{\begin{array}{ccc} \frac{\lambda^{n}}{n!\mu^{n}}P_{0} & \text{if} & l\leq n<c\\ \frac{\lambda^{n}}{c^{n-c}c!\mu^{n}}P_{0} & \text{if} & c\leq n<K \end{array}$$

(15) $$P_{0}=\{\begin{array}{ccc} {\left(\sum_{n=0}^{c=1}\frac{r^{n}}{n!}+\frac{r^{c}}{c!}\frac{1-\rho^{K-c+1}}{1-\rho }\right)}^{-1} & \text{if} & \rho \neq 1\\ {\left(\sum_{n=0}^{c=1}\frac{r^{n}}{n!}+\frac{r^{c}}{c!}(K-c+1)\right)}^{-1} & \text{if} & \rho =1 \end{array}$$

Besides, we have to calculate the average length of each *Notify queue* in order to estimate the overhead

SIP-Event-Notify messages:

(16) $$L_{q}=\frac{P_{0}r^{c}\rho }{c!{\left(1-\rho \right)}^{2}}[1-\rho^{k-c+1}-(1-\rho )(K-c+1)\rho^{K-c}]$$

(17) $$\begin{array}{ccc} L=L_{q}+r(1-P_{K}), & W=\frac{L}{\lambda (1-P_{k})}, & W_{q}=\frac{L}{\lambda (1-P_{k})}-\frac{1}{\mu } \end{array}$$

For Equations (14)–(17) we define ${\lambda =\lambda }^{t}N_{S}^{t}$ and $c=N_{N}^{t}$. Eventually, according to [34] and using Equation (17), the average SIP-Event-Notify messages to subscribe to *m* resources and receive the corresponding notifications (irrespectively of the number of resources that the entity subscribes to) can be defined as:

(18) Average_SIP-Event-Notify_messages=6+2L

## 5. Validation

With the aim to evaluate our proposal, on the one hand we have carried out a proof-of-concept focused on validating the delivery process (over SAML) of security data and information related to the different events that are happening in the system. The *sleepyhead-based* delegation protocol operation, the *Privacy Engine* and the interactions among different entities of our system have been examined as well for correctness. In this way, we can prove the feasibility of our proposal and make easier the determination of deployment requirements.

On the other hand, we considered it necessary to perform some simulations to estimate the SIP-Event-Notify message overhead generated during the system operation. This is very important in our approach, because it is the main extra part that has been added to the system. The performance of credential issuing is not relevant with regard to time-based credential issuing systems, as so far we do not have a sufficiently rich ecosystem or a large number of entities to conduct a real experiment. To this end, we have simulated an event engine through Matlab/Simulink tool [35], including various event sequences with different arrival rates and service times in a random way.

It is worth to mention that a quantitative validation in order to show the advantages obtained with respect to time-dependent revocation solutions has not been performed, because of the scarce deployment of consent management systems that avoids to get mean values (e.g., mean time of credential validity) to do a fair comparison. Although metrics are not defined yet in this area and metrics used for PKC revocation [36] do not apply directly to our proposal, we could use metrics as credential issuing overhead, revocation list management cost, *etc.* These metrics can be calculated without taking into account qualitative advantages, such as credentials being expired when these would be required, for example. In addition, metrics related to delegation chain length are considered as future line.

### 5.1. Health Care Application Scenario

Let us start with some naming conventions for our health care scenario. We will use the term IdP for entities archiving medical records, and with the term SP we refer to consumers of the medical records: hospitals, ambulances and even individuals (doctors). Alice can authenticate by means of a credential to the hospital that persists her medical history (*i.e.*, IdP_1_). In case of an accident, IdPs will provide access to SPs to Alice's medical record (or part of it), according to the events. SPs should demonstrate to IdPs, by means of any sort of authentication, that they are eligible (trusted entity as a hospital) to access medical records.

In our scenario, Alice suffers an accident that triggers several events. The emergency service or 911 (SP_1_) requests access to Alice's medical records in order to send them to an ambulance company (SP_2_) in another trusted domain, which needs access to her medical records to provide Alice the appropriate treatment. Thus, as events happen, they are notified to the involved parties, such as the medical record service (IdP_1_) and the ambulance (SP_2_) which treats Alice. So the IdP_1_ may know which ambulance should be allowed to access to medical histories.

**Figure 4.**
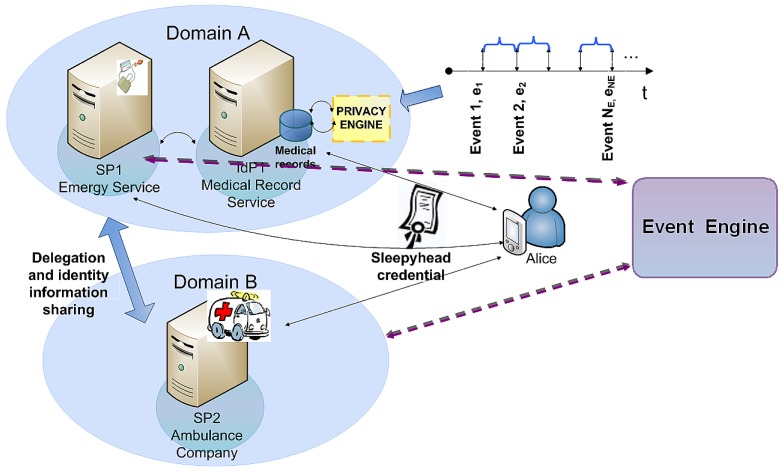
Health care event-based scenario across different domains.

Furthermore, every medical record access request should be related to an event and bound to a purpose, which enables the IdP to filter the access to certain parts of medical history according to a policy. Thus, in this example the following events could be distinguished:

- *Event 1*: There is an accident. SP_1_ notifies this event and calls all ambulance services close to the area.
- *Event 2*: SP_2_, that is subscribed to SP_1_ events, arrives on the scene and requests access to Alice's medical history. It must give a description of the severity of the problem (event) to allow IdP_1_ to give access to certain parts of Alice's medical records or her full history to treat her (purpose). To illustrate this, consider that Alice has broken her femur, has lost her consciousness and needs surgery. In this case, access to the whole medical record could be provided. However, if the problem is minor, as a sprained ankle, SP_2_ is allowed to access only to trauma and drug allergies sections of the history.
- *Event 3*: Although not depicted in the Figure 4, another possible event would be fired if Alice is taken to hospital (SP_3_). The hospital diagnoses her with trauma during the triage and determines that Alice requires an operation. Therefore, a doctor belonging to the hospital (SP_3_) could read Alice's records.

It must be noted that events may be fired by authorized entities, like the emergency service or a hospital urgency service. Likewise, events happen asynchronously and the duration of each event lasts from the beginning of the event itself (*T*_1_) until another event arrives (*T*_2_) whose circumstances and context have changed; and it may contain new requested attributes or privileges. Thus, certain attributes or privileges previously granted will be deactivated and new components of the *sleepyhead* credential will be activated.

### 5.2. Implementation Details

We have deployed our own identity management infrastructure using Lasso [37], a C library which implements the full SAML 2.0/ID-FF stack. The IdPs of the systems are based on Authentic [38], a software that has been developed from Lasso. This library uses OpenSSL as the underlying cryptographic library and Apache2 as the web server. Regarding SPs, we have used ZXID [39]. Furthermore, with the aim to simulate the system of medical events through the *SIP-Notify-Event* specification, we have deployed a Sailfin Application Server [40] and implemented a set of modules that handle the associated logic to subscribe or register events as well as send appropriate notifications to each of the participating entities. Moreover, we have deployed a Registrar Server to ensure participants are authenticated and registered before exchanging any subscription or notification messages. Once registered, entities might exchange messages containing an Event header that indicates the event type to which the entity is subscribed. As for the Expire header, it specifies subscription duration. Finally, event descriptions are sent through XML messages embedded in SIP requests.

**Figure 5.**
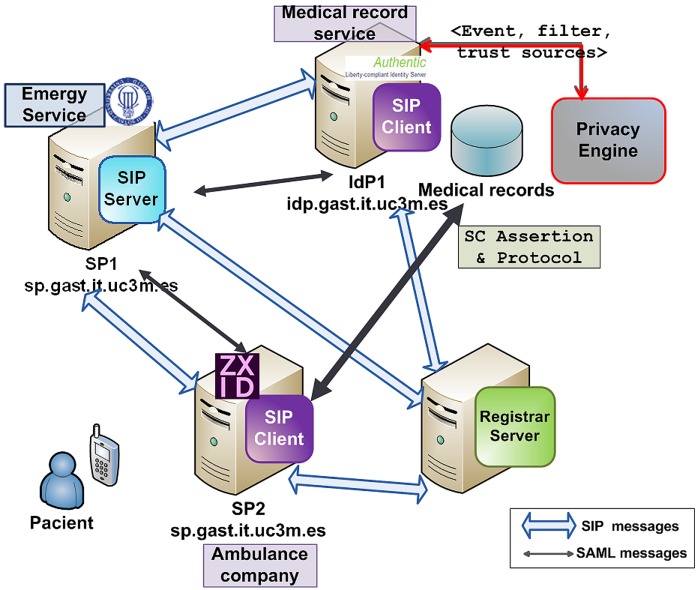
Test architecture. It can be seen the different interactions between the entities (an IdP and two SPs) through the exchange of SIP and SAML messages. Firstly, the SIP clients are registered in the Registrar Server by sending REGISTER messages. Then, the SIP clients subscribe to different events by means of SUBSCRIBE Requests. The SIP Server notifies events to the subscribed entities through NOTIFY Responses. Once events are received, they are analyzed by the Privacy Engine and the sleepyhead credentials are exchanged through SAML requests and responses.

The identity management architecture used for the experiments is depicted in Figure 5. We used that architecture to introduce the modifications proposed in Section 4. For this purpose, we developed a preliminary version of the *Privacy Engine* module that we are currently refining, which is able to receive a data structure that represents the event filter and a hash table that contains the event sources. This building block is in charge of checking that event notifiers are in its *Dynamic Trust List* (DTL) [16] and accomplishes the decision making process that would determine under which conditions or restrictions can the attributes of the medical record be disclosed or the privileges be activated. Since the IdP is the entity handling the medical records as well as the credentials, the *Privacy Engine* operation is closely related to the IdP. Thus, the *Privacy Engine* in our solution is an addition to the original IdP.

Besides, we have extended the Lasso library defining a new structure that represents the *sleepyhead credential* SAML assertion, as well as its different fields and associated attributes. Such assertion is exchanged through SAML messages. To do this, we have modified the metadata exchanged by the IdP and the SPs in order to include the URL or the endpoints to which the *sleepyhead credential* messages would be sent or from they would be received, *i.e.*, the location of the Sleepyhead Credential Consumer Service. In addition, it must be noted that the SP and IdP have been extended by implementing the SAML-based delegation protocol. Thus, we are currently working in order to integrate the new software components with the SIP-based event system to offer a really enhanced privacy experience and to apply audit services for events.

### 5.3. Simulation of the Event Engine

In this section we present and analyze initial results of the simulations carried out in order to calculate the SIP-Notify-Event messages overhead and to demonstrate our system robustness and scalability. Firstly, we have simulated an event engine by using the MATLAB mathematical tool, which generates generic health care events that arrive to an *M/M/c/K* notification queueing system and they are served as described in Section 4.4. For this purpose, we used a set of statistical data collected by the HES (*Hospital Episode Statistics*) online service [41], which reflects basic information related to patient arrival rates and waiting times within Emergency Departments of the main hospitals in England.

**Figure 6.**
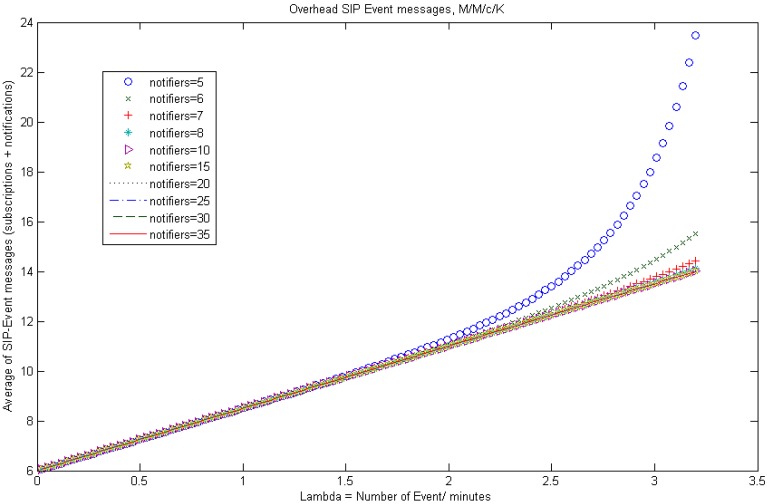
Overhead SIP-Event Notify messages by varying the notification arrival rates and the number of notifiers.

Figure 6 represents several plots of Equation (18) for an scenario composed by 10 subscribed entities and distinct number of notifiers (from 5 to 35), with 1/*μ* =1.25 events per minute, *K* = 100 and different event arrival rates. As it is shown in Figure 6, the event engine based on the SIP-Specific-Event-Notify protocol introduces an assumable message overhead, which grows linearly with the arrival frequency of events to the system. It must be noted that, in the performed simulation the message overhead starts to grow exponentially when the event arrival rate doubles the notifiers. This is clearly seen for 5 notifiers; therefore, the parameter that represents congestion of the system is approaching to its saturation value (*ρ* ≈ 1). So, it will be necessary to establish a trade-off between the number of notifiers that serve the different type of events, the number of entities subscribed to them and the maximum size of the queue for each event type, in order to avoid loss of messages without saturating the system.

**Figure 7.**
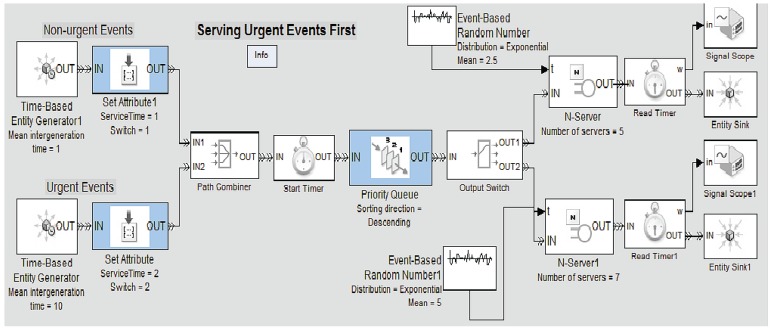
Multi-server notification queueing system that serves urgent events first. Example of Simulink block diagram.

Currently, we are working on improving the event engine, by defining a Simulink model that enables to represent our multi-server notification queueing system in a more realistic manner. Basically, this model consists of the following components: Time-based Generators, Priority Queues, Event-based random Generators and Servers acting as notifiers. Regarding the priority queues, our system only classifies the simulated health care events into two categories: “Urgent Events” and “Non-urgent Events”. Thus, the system will serve first the events assigned with higher priority. Figure 7 illustrates an example of the architecture of the multi-server notification queueing model. Furthermore, we aim to use a more complete data set of the accident and emergency (A&E) attendance within HES, which covers the period from April 2010 to March 2011 [42], in order to take into account aspects related to emergency attendances and average times in the different patient's treatment phases (*i.e.*, primary diagnosis, admission, diagnosis, treatment, *etc.*), depending on the month, day, hour of arrival, *etc.* Further research could be done to contemplate more event categories and define new priority levels.

## 6. Related Work

As far as the related work is concerned, due to the increasing interest in e-health applications, security and privacy are becoming important concerns for researchers. However, there is still scarce work on effective revoking consent mechanisms within health care environments. The approach presented in [43] is close to our work. The authors propose an activity-oriented access control model to protect the confidentiality of health information, which consists of three levels: user level, activity level and privilege level. Thus, this proposal is based on user activity to authorize access privileges and defines two revocation mechanisms called single-step and multi-step revocations. However, possible activities in the hospital and policies need to be defined in advance. In [44], a privacy-aware role-based access control (P-RBAC) is presented, in order to express privacy policies. These policies are seamlessly integrated with access control. In this same way, paper [45] presents the notion of consent and revocation policies to express user's preferences, within the context of the EU FP7 EnCoRe project [46]. This work has been extended in [47] and [48] by proposing a conceptual model for privacy policies that can be integrated with XACML (eXtensible Access Control Markup Languages). They also consider revocation of personal data as well as previously granted privileges. Nevertheless, these works propose traditional revocation mechanisms such as temporal information being used to predefine policies. They do not take into account dynamic scenarios or information systems designed for emergency situations.

## 7. Conclusions and Future Work

We have reviewed and analyzed the main identity models and current frameworks to preserve privacy in identity management systems, identifying its main lacks and drawbacks. Specifically, in this work we have addressed the relevant issue of effective consent revocation, since it is covered by none of the analyzed IdM technologies and it is a must-have requirement in sensitive environments, such as health care scenarios. Current approaches are focused on temporal information-based revocation mechanisms being used to predefine policies. Thus, we have proposed a hybrid IdM event-driven model, which includes a *sleepyhead credential*-based delegation protocol compliant with the SAMLv2 standard to provide a more flexible user consent-revocation mechanism within health care scenarios. The main advantages are that this credential is issued only once and would be used any time; while time-based credentials have to be periodically re-issued, for short windows of time to minimize unauthorized accesses, as required. Note that, a quantitative validation showing the advantages achieved has not been provided, due to the scarce deployment of consent management infrastructure that avoids to obtain mean values to perform a fair comparison.

Our solution proposes using events to wake up dormant privileges or part of them and it incorporates new features that allow better scalability, since the emergency services are the entities which manage trust indirectly. Moreover, in this paper we have presented a mathematical model based on Markov's chains and theory queues to determine and study different health care event arrivals to the system and how they are handled and notified to the corresponding subscribed entities. To validate our solution, on the one hand, we have implemented a proof-of-concept focused on testing the operation of the *sleepyhead credential*-based delegation protocol and the *Privacy Engine*. On the other hand, we have applied the aforementioned mathematical model to estimate the SIP-Event-Notify message overhead. Initial results show that the event engine introduces an assumable message overhead. However, important parameters such as the number of notifiers and the maximum size of the notification queue must be controlled in order to avoid loss of messages without saturating the system. In addition, it must be noted that the usage of the system also affects privacy and should be present in users consents. The auditing processes should verify that the design and assumptions regarding future usage match its actual usage.

For future work, we want to test this last issue on real health care scenarios in order to demonstrate how the privacy is managed by the system actors. Likewise, further analysis is needed in the topic of self-revocation of sleepyhead credentials. We also plan to take into account different privacy requirements for identity attributes, including biometric and health care data. Further research is also needed in preserving user privacy during the exchange and sharing of attributes in different trust domains, also considering usability of the system. Finally, as immediate future lines with regard to the event engine, we plan to study in-depth aspects related to emergency attendances and average waiting times in the different patient's treatment phases to tackle more complex event arrival patterns.

## Figures and Tables

**Table 1.** Summary of privacy features in Identity Management.

**IdM Technology**	**Anonymity and Pseudonimity**	**Unlinkability and unobservability**	**Privacy Languages**	**Revoking consent**
Federated Model (SAML/ID-FF)	Partial anonymity (IdP knows user identity). No solution from preventing IdPs from tracking is provided.	Transient and permanent identifiers. Different pseudonyms for each SP recommended. Confidentiality of transaction recommended. Cryptographic mechanisms do not prevent from traffic analysis attacks.	The XSPA-SAML profile enables to obtain user's consent and describe attributes to preserve privacy in health care. An identity governance framework is defined.	**Not addressed**
User-centric Model (InfoCards)	Included in the specification	Message flow eliminates direct communication IdP-SP. Identity selector may encrypt SP identity to prevent the IdP from learning.	Allows to express privacy policies of RPs.	**Not addressed**
Hybrid Model (OpenID)	Not addressed	Not addressed	Not addressed	**Not addressed**

**Table 2.** Definition of the parameters for the event model.

**Parameter**	**Definition**
*N_ES_*	Total number of entities in the system
*N_E_*	Number of possible event types (matches the Markov's chain states)
*N_N_*	Number of notifier entities in the system
*N_S_*	Number of subscriber entities in the system,
$N_{N}^{t}$	Number of notifiers in the system delivering events of type *e_t_*
$N_{S}^{t}$	Number of entities subscribed to events of type *e_t_*
$p_{N}^{t}$	Percentage of notifiers in the system delivering events of type *e_t_*
$p_{S}^{t}$	Percentage of entities subscribed to events of type *e_t_*
*M^t^*	Message size to be transferred, considering the overhead introduced by the protocol, when an *e_t_* event is delivered
λ*^t^*	Rate of *e_t_* event arrival
λ*^t,k^*	Rate of *e_t_* event notification rate for notifier *n_k_*
$\lambda_{j}^{t}$	Rate of arriving events of type *e_t_* to entity *e_j_*
$\lambda_{j}^{t,k}$	Rate of notified events of type *e_t_* by the *n_k_* notifier that arrives to the entity *e_j_*
$P_{en,j}^{t}$	Percentage of notified events of type *e_t_* by the *n_k_* notifier that arrives to the entity *e_j_*
*μ*	Service time for notification of events of type *e_t_*
*c*	Number of servers or notifiers attending notification of *e_t_*
*ρ* = λ/(*cμ*)	Congestion of the system with parameters λ, *μ* and *c*
*K*	Maximum number of notification messages that can be buffered by the queue
*P_N_*	Probability of having *n* notification messages in the system
*P*_o_	Probability of having 0 notification messages in the system
*L_q_*	Notification message queue size
*L*	Average notification messages in the system
*W_q_*	Average waiting time of notification messages in the queue

## References

[1] Tentori M., Favela J., Gonzlez V.M. (2006). Quality of Privacy (QoP) for the Design of Ubiquitous Healthcare Applications. J. Univ. Comput. Sci..

[2] Qiu Y., Zhou J., Baek J., Lopez J. (2010). Authentication and Key Establishment in Dynamic Wireless Sensor Networks. Sensors.

[3] Kumar P., Lee H.J. (2011). Security Issues in Healthcare Applications Using Wireless Medical Sensor Networks: A Survey. Sensors.

[4] Jara A., Zamora M., Skarmeta A. (2011). An Internet of Thingsbased Personal Device for Diabetes Therapy Management in Ambient Assisted Living (AAL). Person. Ubiquit. Comput..

[5] Ryan W.G. (1967). Privacy and Freedom: Alan F. Westin Atheneum Publishers. Business Horizons.

[6] Gunn P.P., Fremont A.M., Bottrell M., Shugarman L.R., Galegher J., Bikson T. (2004). The Health Insurance Portability and Accountability Act Privacy Rule: A Practical Guide for Researchers. Med. Care.

[7] Looby J. (2010). Discovering Europe: How to Navigate Europe's Privacy Protections. The National Law Journal.

[8] (2009). EU Directive 2002/58 on Privacy and Electronic Communications (E-Privacy Directive). Amended by Directive 2009/136. Official J. EU.

[9] Curren L., Kaye J. (2010). Revoking Consent: A Blind Spot in Data Protection Law?. Comput. Law Secur. Rev..

[10] Pfitzmann A., Khntopp M. (2001). Anonymity, Unobservability, and Pseudonymity—A Proposal for Terminology. Lect. Notes Comput. Sci..

[11] The Common Criteria Project Sponsoring Organizations (2007). Common Criteria for Information Technology Security Evolution. http://www.commoncriteriaportal.org/files/ccfiles/CCPART2V3.1R2.pdf.

[12] Jøsang A., Zomai M.A., Suriadi S. Usability and Privacy in Identity Management Architectures.

[13] Hirsch F., Philpott R., Maler E. (2005). Security and Privacy Considerations for the OASIS Security Assertion Markup Language (SAML) V2.0.

[14] Cooper D., Santesson S., Farrell S., Boeyen S., Housley R., Polk W. (2008). Internet X.509 Public Key Infrastructure Certificate and Certificate Revocation List (CRL) Profile.

[15] Liberty Alliance Project Liberty ID-FF Protocols and Schema Specification. http://www.projectliberty.org.

[16] Arias Cabarcos P., Almenrez Mendoza F., Marn-Lpez A., Daz-Snchez D. (2009). Enabling SAML for Dynamic Identity Federation Management. IFIP Adv. Inform. Commun. Technol..

[17] Information Cards Information Cards Foundation. http://informationcard.net/.

[18] Nanda A., Jones M.B. (2008). Identity Selector Interoperability Profile V1.5. http://www.identityblog.com/wp-content/resources/2008/Identity_Selector_Interoperability_Profile_V1.5.pdf.

[19] XSPA Profile Cross-Enterprise Security and Privacy Authorization (XSPA) Profile of Security Assertion Markup Language (SAML) for Healthcare Version 1.0. http://docs.oasis-open.org/security/xspa/v1.0/saml-xspa-1.0-cs01.html.

[20] Liberty Alliance Project An Overview of the Id Governance Framework. http://projectliberty.org/liberty/content/download/3500/23156/file/overview-id-governance-framework-v1.0.pdf.

[21] Liberty Alliance Project Privacy Preference Expression Languages. http://www.projectliberty.org.

[22] Pitzman B. (2004). Privacy in Enterprise Identity Federation Policies for Liberty 2 Single Sign on.

[23] Recordon D., Jones M., Bufu J., Daugherty J., Sakimura N. OpenID Provider Authentication Policy Extension 1.0. http://www.openid.net.

[24] Mouttham A., Peyton L., Eze B., Saddik A. (2009). Event-Driven Data Integration for Personal Health Monitoring. J. Emerg. Technol. Web Intell..

[25] Baarah A., Mouttham A., Peyton L. Improving Cardiac Patient Flow Based on Complex Event Processing.

[26] Roach A.B. (2002). Session Initiation Protocol (SIP)—Specific Event Notification.

[27] McCarthy M.L., Zeger S.L., Ding R., Aronsky D., Hoot N.R., Kelen G.D. (2008). The Challenge of Predicting Demand for Emergency Department Services. Acad. Emerg. Med..

[28] Rathlev N.K., Chessare J., Olshaker J., Obendorfer D., Mehta S.D., Rothenhaus T., Crespo S., Magauran B., Davidson K., Shemin R., Lewis K., Becker J.M., Fisher L., Guy L., Cooper A., Litvak E. (2007). Time Series Analysis of Variables Associated With Daily Mean Emergency Department Length of Stay. Ann. Emerg. Med..

[29] Schull M.J., Kiss A., Szalai J.P. (2007). The Effect of Low-Complexity Patients on Emergency Department Waiting Times. Ann. Emerg. Med..

[30] (1984). Modeling and Analysis of Computer Communications Networks.

[31] Cantor S. (2009). SAML V2.0 Condition for Delegation Restriction. http://docs.oasis-open.org/security/saml/Post2.0/sstc-saml-delegation-cd-01.pdf.

[32] Sato R.C., Zouain D.M. (2010). Markov Models in Health Care. Einstein Sao Paulo.

[33] Gross D., Shortle J.F., Thompson J.M., Harris C.M. (2008). Fundamentals of Queueing Theory.

[34] Zhao B., Liu C. Efficient SIP-Specific Event Notification.

[35] MathWorks, Using MATLAB Manual. http://www.mathworks.com/.

[36] Nigusse G.E. (2007). Evaluating Public Key CertificateRevocation Schemes: Towards ConceptuallyVersatile Revocation Scheme. M.S. Thesis.

[37] Lasso: Liberty Alliance Single Sign-On. http://lasso.entrouvert.org/.

[38] Authentic: Liberty-Compliant Identity Provider. http://authentic.labs.libre-entreprise.org/.

[39] SymLabs.: ZXID: Open SAML Implementation in C. http://www.zxid.org/.

[40] Project Sailfin: Open source Java Application Server Project. http://sailfin.java.net/.

[41] The Information Center for Health and Social Care: Statistics & Data Collections. http://www.ic.nhs.uk/statistics-and-data-collections.

[42] Accident and Emergency Hospital Episode Statistics (HES). http://www.ic.nhs.uk/statistics-and-data-collections/hospital-care/accident-and-emergency-hospital-episode-statistics-hes.

[43] Hung L.X., Lee S., Lee Y.K., Lee H. Activity-Based Access Control Model to Hospital Information.

[44] Ni Q., Trombetta A., Bertino E., Lobo J. Privacy-Aware Role Based Access Control.

[45] Whitley E.A. (2009). Informational Privacy, Consent and the “Control” of Personal Data. Inf. Secur. Tech. Rep..

[46] EnCoRe (*Ensuring Consent & Revocation*) European Community's Seventh Framework Programme [FP7/2007-2013]. http://www.encore-project.info/.

[47] Agrafiotis I., Creese S., Goldsmith M., Papanikolaou N., Mont M.C., Pearson S. Defining Consent and Revocation Policies.

